# Implication of Soluble *HLA-G* and *HLA-G +3142G/C* Polymorphism in Breast Cancer Patients Receiving Adjuvant Therapy in Tanzania

**DOI:** 10.31557/APJCP.2019.20.11.3465

**Published:** 2019

**Authors:** Ismael Chatita Adolf, Gokce Akan, Teddy F Mselle, Nazima Dharsee, Lucy A Namkinga, Fatmahan Atalar

**Affiliations:** 1 *Department of Biochemistry, MUHAS Genetics Laboratory, Muhimbili University of Health and Allied Sciences, *; 2 *University of Dar es Salaam, Mbeya College of Health and Allied Sciences, *; 3 *Ocean Road Cancer Institute, Tanzania, *; 4 *4Child Health Institute, Department of Medical Genetics, Istanbul University, Turkey. *

**Keywords:** Breast cancer diagnosis, soluble HLA-G, HLA-G +3142G/C, mastectomy, Tanzanian population

## Abstract

**Background:**

During cancer growth, immunosuppressive microenvironment is created that enables tumour cells to evade an eliminative immune response and hence manage to grow into malignancy. HLA-G, existing as either membrane-bound (mHLA-G) or soluble (sHLA-G) molecule is thought to be immunosuppressive and produced more by tumor cells. The +3142G/C polymorphism in *HLA-G* gene affects its expression, and G allele is considered to be a protective mutant allele associated with less expression of HLA-G. The implication of HLA-G in cancer development has been reported in different cancers and populations. But, its implication in most African populations has not yet been investigated. The aim of this study was to determine the possible associations of soluble HLA-G and HLA-G +3142G/C SNP with breast cancer.

**Materials and Methods:**

75 breast cancer patients and 84 normal controls were recruited in this study. The genotyping of HLA-G +3142G/C polymorphism was determined by LightSNiP typing assay using quantitative Real-Time PCR and sHLA-G levels were determined by ELISA.

**Results:**

The sHLA-G levels were significantly lower in breast cancer patients than in controls (p<0.001). Also, they were significantly lower in mastectomized patients compared to non-mastectomized patients (p=0.018). The ROC analysis revealed a significant ability of sHLA-G to differentiate breast cancer patients versus normal controls (AUC=0.697, 95% CI= 0.619-0.767, p<0.001) and identify mastectomized patients (AUC=0.667, 95% CI= 0.549 to 0.772, p=0.041). The assessment of +3142G/C polymorphism revealed a relatively similar distribution of frequencies of genotypes and alleles between breast cancer patients and normal controls (p>0.05) and was neither associated with sHLA-G levels.

**Conclusion:**

While the +3142G/C SNP was found not to be relevant to breast cancer, the changes of sHLA-G levels in response to medical interventions such as mastectomy may be translated into its potential prognostic utility for breast cancer. More studies are needed to provide clear evidence of sHLA-G as a diagnostic and prognostic marker of breast cancer in Tanzania.

## Introduction

The implication of HLA-G in various pathological conditions is increasingly becoming evident. Through its immunomodulation functions, it has been reported to play role in pathogenesis of various cancers (Loumagne et al., 2014; Zhang et al., 2016; Caocci et al., 2017; Kowal et al., 2015) autoimmune diseases (Hachiya et al., 2016) viral (Yan, 2014) and parasitic infection (Sabbagh et al., 2018). It also has been associated with pregnancy complications such as pre-eclampsia, recurrent abortions (Nilsson et al., 2014) and even in determining transplantation success (Rouas-Freiss et al., 2003).

HLA-G is a non-classical HLA-1 molecule with immunosuppressive properties. There are seven isoforms of HLA-G protein classified into membrane bound (HLA-G-1, G-2, G-3, G-4) and soluble (HLA-G-5, G-6, G-7) isoforms (González et al., 2012). The isoforms result from alternative splicing of the primary transcript of *HLA-G* gene. However, the soluble forms can also be produced from proteolytic cleavage of membrane bound HLA-G from cell surface and its subsequent release in the circulatory system (Alegre et al., 2014).

At a normal physiological state, HLA-G is preferentially expressed in immune privileged sites such as thymus, cornea, pancreatic islets, and in cells of hematopoietic lineage (Curigliano et al., 2013). The immunosuppressive characteristics of HLA-G were firstly discovered in trophoblasts as the molecule that protects a semi-allogeneic fetus from maternal immune attack (Rouas-Freiss et al., 1997). And its deregulated expression was further found to be implicated in pathological conditions such as those aforementioned above.

The sHLA-G has been explored in context of its potential utility for diagnosis and prognosis of various cancers. An increased level of sHLA-G has been observed in breast cancer (Chen et al., 2010), prostate cancer (Heidari et al., 2017), colorectal, gastric, esophageal and lung cancers (Cao et al., 2011) patients. Some other studies have associated HLA-G with cancer progression, revealed by higher expression level of HLA-G in high stage and metastasized cancers than in low stages and non-metastasized cancers (Gimenes et al., 2014; Li et al., 2012; Goncalves et al., 2016; Kirana et al., 2017).

However, some medical interventions can bring about changes in sHLA-G levels among cancer patients. Chemotherapy has been reported to reduce the plasma level of sHLA-G in ovarian cancer (Rutten et al., 2014). Also, breast cancer surgery followed by adjuvant therapy has been reported to significantly reduce sHLA-G below the physiological level (Sayed et al., 2010). 

While there is a little variability in HLA-G coding region and protein, both promoter and 3’ untranslated (3’UTR) regions bear polymorphisms which affect the levels of *HLA-G* expression (Castelli et al., 2011; Martelli-Palomino et al., 2013), and some have been associated with different pathologies such as cancer and autoimmune diseases (Hachiya et al., 2016; Rolfsen et al., 2014).

There is however a limited information on how *HLA-G* is implicated in various cancers in African population. The aim of this study was to investigate whether s*HLA-G* level and *HLA-G +3142G/C SNP* are associated with breast cancer in Tanzanian patients.

## Material and Methods

*Study population*

Breast cancer patients at Ocean Road Cancer Institute (ORCI) were recruited in this study. Women attending the breast cancer, cervical cancer and HIV screening center at ORCI were recruited as normal controls, provided that the screening results for both conditions were negative. The total of 159 women, 75 breast cancer patients and 84 normal controls participated in this study. 

The patients’ information relevant to this study was sought from their medical files upon being approved by ORCI medical information manager. The collected information included age, ethnicity, mastectomy status and metastatic status. Also, the data included the dates on which mastectomy was performed and when metastasis was reported following mastectomy.

The written consent was sought before taking biological samples from patients and clinical information from their files. This study was approved by Muhimbili University of Health and Allied Sciences ethical committee. The ethical clearance letter was issued on 17th April, 2018 with the reference number DA.287/298/01A/. 

*Quantitative determination of plasma sHLA-G levels*

The peripheral blood was collected from arms of the patients into red cupped blood collecting tubes. The blood was subsequently centrifuged and the plasma was collected and stored at -200C until the day ELISA was performed. 

ELISA kits (purchased from Qayee Biological Technology Co., Ltd, Shanghai, China) were used for quantitative determination of *sHLA-G* in plasma from patients and controls. The plasma samples were five times diluted by initially adding 40µl of diluent followed by 10µl of plasma samples into designated wells of 96-welled ELISA plate pre-coated with monoclonal antibody. This was followed by the addition of 50µl of horseradish peroxidase conjugate solution and incubated for 1 hour at 370C. After that incubation time, the contents were discarded and the plate was washed five times. Thereafter, chromogen solutions A and B were added and waited for 10 minutes after which stop solution was added. The optical densities (ODs) resulting from color change were read at 450 nm wavelength under ELISA reader. The ODs were converted into their corresponding concentrations using the ELISA analysis software obtained at elisaanalysis.com.

*DNA extraction*

DNA was extracted from peripheral blood collected into 10 ml EDTA tubes. DNA was manually extracted using Wizard® Genomic DNA Purification Kit (Promega, Madison, USA) according to the instructions of the manufacturer. The quantity and quality of extracted DNA were determined by the NanoDrop™ 1000 Spectrophotometer (Thermo Fisher Scientific, Waltham, MA, USA). The DNA was stored at -20˚C until the day of experiment.

*Genotyping of HLA-G +3142G/C and data analysis*

Both controls and patients were analyzed for the *HLA-G +3142 G/C (rs1063320)* by the use of quantitative real time PCR (LightCycler® 480 system, Roche-Germany). Genotyping was achieved through LightSNiP typing assay (TIBMolBiol, Berlin, Germany) and subsequent melting curve analysis. 

The data for allelic and genotypic frequencies of *HLA-G +3142G/C* in African related populations were downloaded from 1000 Genomes project phase-3 database (https://www.ncbi.nlm.nih.gov/variation/tools/1000genomes/.) and HapMap project (https://www.snpedia.com/index.php/Rs1063320). The genetic data obtained from HapMap were of Luhya in Webuye from Kenya (LWK), Maasai in Kinyawa from Kenya (MKK) and people of African ancestry living in Southwest USA (ASW). Those obtained from 1000 Genomes represented Yoruba population from Nigeria (YRI) and sample generalizing all African population (AFR).

*Statistical analysis*

Non-parametric statistical tests were used to compare the levels of *sHLA-G* between the groups. Mann-Whitey was used to compare the *sHLA-G* levels between two groups, while Kruskal-Wallis test was used to compare *sHLA-G* levels between more than two groups. Kaplan-Meier test was used to test how strongly a risk allele can predict the metastatic free survival of patients following mastectomy. All these aforementioned tests were contained in Statistical Package for the Social Sciences (Statistical Package for the Social Sciences, SPSS Inc, Chicago, IL, USA, version 25). The Receiver Operating Characteristic (ROC) Curve Analysis test contained in MedCalc software (MedCalc Software, Mariakerke, Belgium) was used to assess the diagnostic and prognostic utility of *sHLA-G*. Chi-square (x^2^) was used for comparison of allelic and genotypic frequencies distributions of +3142G/C between the two populations. The test for conformity of populations’ genotypic frequencies distributions to Hardy Weinberg (HWE) expectation was done by Fisher’s test. The two tests were integral parts of HWE software. All the tests were considered to be statistically significant when p<0.05.

## Results

*Clinical characteristics of study population*

A total of 75 breast cancer patients and 84 controls participated in this study. The mean ages of patients were 51.04±12.54, while that of controls were 43.36±11.64. Table 1 summarizes the characteristics of the patients. Most patients were under medical care, being treated with chemotherapy or radiotherapy or hormone therapy or combination thereof. 81.3% of the patients had undergone mastectomy, and most of them had a post-mastectomy time of 12 months. Regarding receptor expression status, the number of patients characterized by negative expression of Progesterone receptor (PR) or Estrogen receptor (ER) or Human Epidermal growth factor receptor (HER2) was higher than that positively expressing such receptors. We could not obtain the receptor expression status of 29.3% of patients from their medical files.

*Plasma Levels of Soluble HLA-G in Breast Cancer Patients and Controls*

The median and mean levels of *sHLA-G* in breast cancer patients were 13.38ng/ml and 23.92ng/ml ± 31.76 respectively. These were significantly lower than median level 22.71ng/ml and mean level 31.58ng/ml ± 28.60, observed in normal controls (p<0.01). Mastectomy was associated with lower levels of *sHLA-G *(p=0.018). No relevance of *sHLA-G* levels was found with respect to receptor expression status (PR, ER and HER2) among breast cancer patients (Table 2).

*Predictive Power and Prognostic Value of sHLA-G Levels*

**Figure 1:**
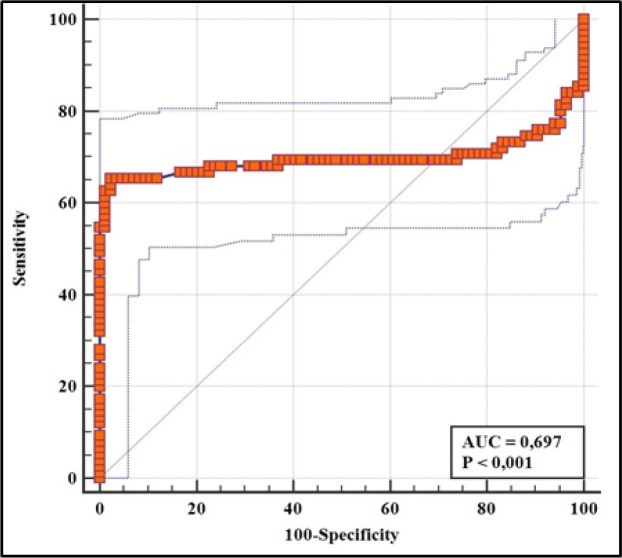
ROC Curve Analysis Assessing the Performance of sHLA-G in Discriminating between Breast Cancer Patients and Controls

**Figure 2:**
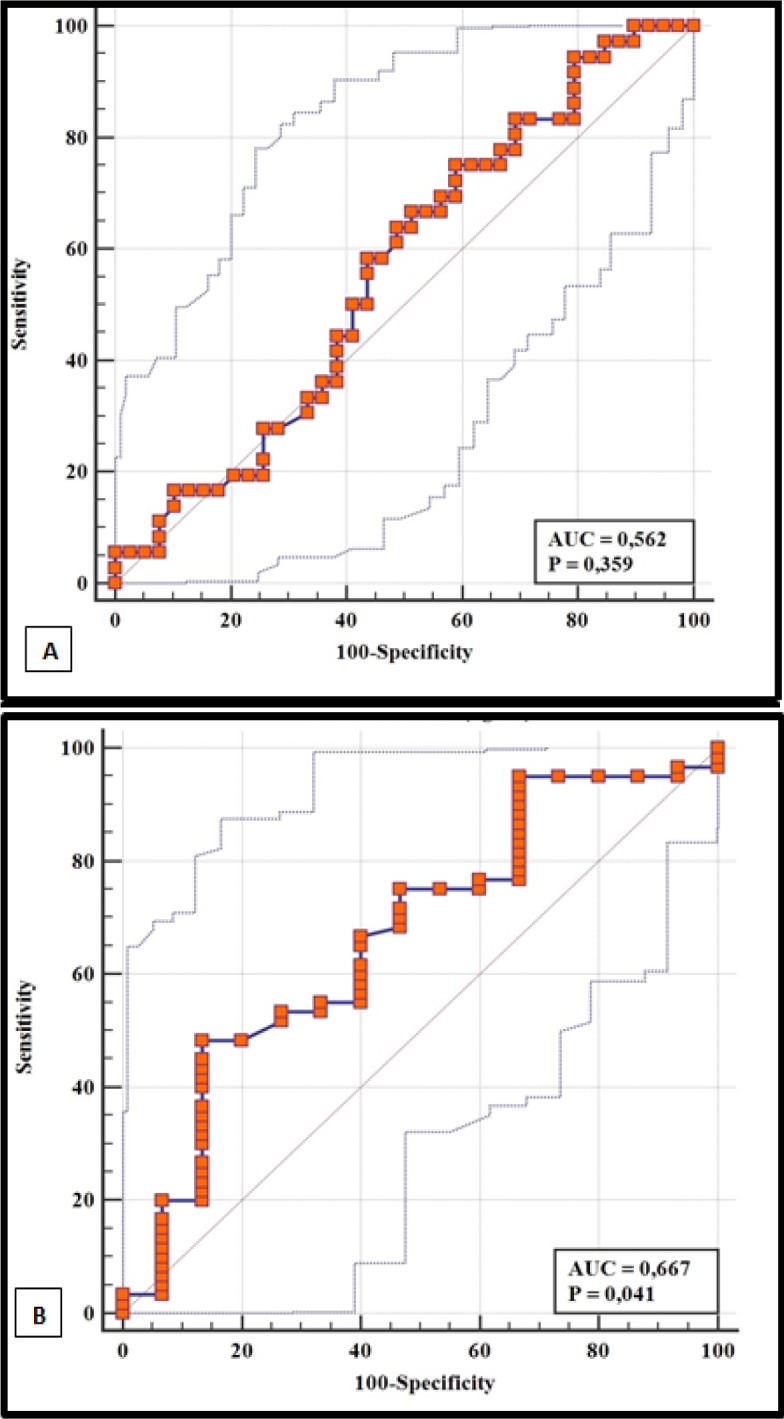
ROC Curve Analysis Assessing the Performance of sHLA-G in Discriminating between A, metastatic and non-metastatic breast cancer patients; B, mastectomized and non-mastectomized breast cancer patients

The ROC curves for predicting breast cancer, metastasis and mastectomy are presented in Figure 1, Figure 2(A) and 2(B) respectively. *sHLA-G* levels demonstrated a good discriminatory power to differentiate between breast cancer patients and normal controls (p<0.001). Taking mastectomy into consideration, a statistical significance was found for *sHLA-G* to identify mastectomized patients (The area under curve (AUC): 0.667, 95 CI: 0.549 to 0.772, p=0.041). The discriminatory power of *sHLA-G* to differentiate between metastatic and non-metastatic breast cancer was poor as *sHLA-G* could not predict metastasis (AUC: 0 .562, 95% CI= 0.442 to 0.676, p<0.35).

*Frequency Distribution of HLA-G +3142G/C Genotypes and Alleles among Breast Cancer Patients and Controls*

The distribution of allelic and genotypic frequencies of *HLA-G +3142G/C* in our study population was summarized in Table 3. The allelic and genotypic frequencies distributions of *+3142G/C *were relatively similar between breast cancer patients and normal controls (p=0.537 and p=0.650 for genotypes and alleles respectively). With G allele as a reference, we could not find any significance in association between +3142G/C SNP and breast cancer (OR=1.26; 95% C.I, 0.6-2.65). In both patients and control groups, the distribution of genotypes was not significantly deviant from the HWE expectation (p=0.09 for controls, p=0.41 for patients).

*HLA-G +3142G/C Genotypes and Plasma Levels of Soluble HLA-G*

**Figure 3:**
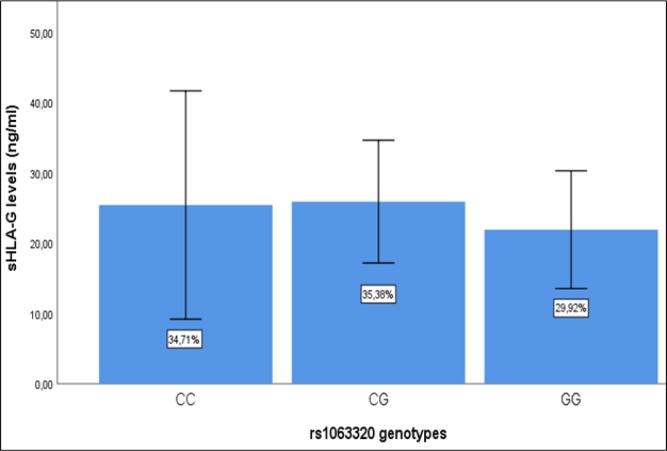
Levels of sHLA-G According to Genotypes in the Study Population *(p=0.684)*

To determine the possible effect of *+3142G/C *on the expression of* sHLA-G*, we determined the association of *+3142G/C* genotypes with *sHLA-G* (Figure 3). The levels of *sHLA-G* across all genotypes were relatively similar (p=0.684). 

*Genotypic and Allelic Frequencies Distribution among Selected African Ethnics*

The data for +3142G/C genotypic and allelic frequencies of African ethnics were extracted from HapMap and 1,000 Genomes projects repositories. The ethnic populations data extracted from HapMap included data for Luhya in Webuye from Kenya (LWK), Maasai in Kinyawa from Kenya (MKK) and people of African ancestry living in Southwest USA (ASW). Data extracted from 1,000 Genomes were of Yoruba from Nigeria (YRI) and sample generalizing all African population (AFR). Their genotypic and allelic frequencies distribution patterns were compared with Tanzanian ones as per genotyping results (TNZ) (Table 4). With the Tanzanian study population as a reference, only Luhya population had a genotype and allelic frequencies distributions relatively similar to it.

*Kaplan-Meier Analysis of Metastasis-free Breast Cancer Survival*

**Figure 4:**
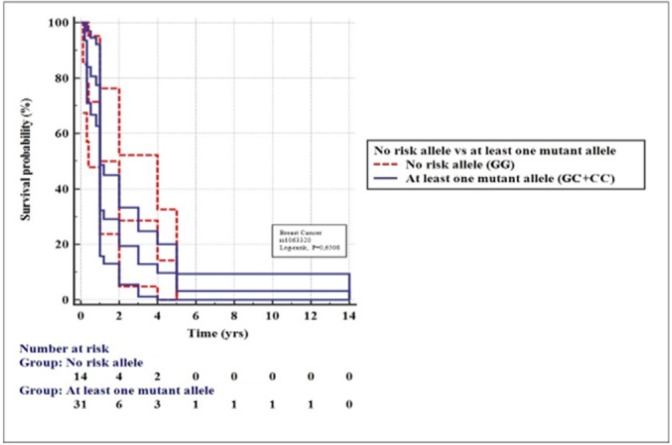
Kaplan-Meier Analysis of Metastasis-Free Survival in Breast Cancer Pients with Mastectomy for +3142G/C Genotypes (*p=0.6508*)

The power of *HLA-G +3142G/C* genetic variants to predict the metastatic-free survival among breast cancer patients was investigated by comparing patients carrying no risk allele (GG) versus those carrying at least one risk allele (CC and CG) (Figure 4). The patients were retrospectively followed up from the date they underwent mastectomy to the date of last news that they had no any metastatic relapse. Metastasis-free survival (MFS) was calculated from the date of diagnosis until the date of first distant relapse using the Kaplan-Meier method. Survival was compared between groups with the log-rank test. In both groups, most metastatic relapses occurred within 5 years following mastectomy. No significant difference in MFS was found between the two groups (Log rank, p=0.6508). 

**Table 1:** Characteristics of Breast Cancer Patients

Characteristics	Study Participants (n=75)
Median Age (IQR)	49 (41-60)
Mean Age (±SD)	51 (±12.54)
< Median age, n (%)	37 (49.3)
≥Median age, n (%)	38 (50.7)
Median BMI (IQR)	26.98 (22.67-33.65)
Mean BMI (±SD)	28.11 (±7.47)
< Median BMI, n (%)	39 (52)
≥Median BMI n (%)	36 (48)
Metastatic status, n (%)	
Metastasized	39 (52)
Non-Metastasized	36 (48)
Mastectomy status, n (%)	
Yes	61 (81.3)
No	14 (18.7)
Receptor status, n (%)	
ER positive	24 (32)
ER negative	29 (38)
PR positive	16 (21.3)
PR negative	37 (49.3)
HER positive	21 (28)
HER negative	32 (42.7)
Unknown	22 (29.3)
Origin, n (%)	
North	21 (28)
South	19 (25.3)
East	19(25.3)
West	6 (8)
Central	10 (13.3)

SD, Standard deviation; IQR, Interquartile range; ER, Estrogen receptor; PR, Progesterone receptor; HER, Human epidermal growth factor receptor

**Table 2:** sHLA-G with Respect to Clinicopathological Parameters

Parameter	sHLA-G levels Mean±SD	sHLA-G levels Median IQR	*p value*
Disease status			
Patients	23.92 ±31.76	13.08 (11.38-18.44)	0.001
Control	25.68±28.60	22.71 (22.29-23.77)	
Mastectomy status			
Yes	21.22±30.64	12.67 (11.29-16.30)	0.018
No	35.70±35.01	15.40 (12.80-55.25)	
Metastatic status			
Metastasized	17.14±15.37	12.93 (11.32-14.80)	0.252
Non-metastasized	30.17±40.79	13.22 (11.64-23.27)	
ER status			
Positive	26.58±41.05	12.76 (11.33-16.84)	0.611
Negative	23.00±31.62	11.77 (11.05-15.40)	
PR status			
Positive	29.91±49.66	12.32 (10.82-16.80)	0.663
Negative	22.34±28.10	12.67 (11.33-15.40)	
HER2 status			
Positive	30.82±43.72	11.44 (10.81-27.87)	0.592
Negative	20.56±29.24	12.76 (11.65-14.84)	

*p-values* were obtained by Mann-Whitney test; Statistical test was significant if *p<0.05*

**Table 3:** Genotype and Allelic Frequency Distribution of +3142G/C among Breast Cancer Patients and Controls: G is a Reference Allele

	*Patients, (%) n=64	**Controls, (%) n=75	X^2^	*p-value*	OR/95%CI
Genotype					
CC	18 (31%)	25 (36.2%)			
GC	32 (55.2%)	27 (39.1%)	0.38	0.537	1.26/0.6-2.65
GG	8 (13.8%)	17 (24.6%)			
Allele					
C	38 (59%)	42 (56%)	0.21	0.65	0.89/0.54-1.46
G	26 (41%)	33 (44%)			

*X^2^, Fitness-of-Fit test for Deviation of genotypic frequency distribution from HWE in Patients; *p=0.420*; **X^2^, Fitness-of-Fit test for Deviation genotypic frequency distribution from HWE in Controls,* p=0.09*; OR, Odd ratio; CI, Confidence interval

**Table 4:** Genotypic and Allelic Frequency Distribution of rs1063320 among African Ethnics

Population		TNZ	LWK	MKK	YRI	ASW	AFR
N		139	89	142	108	49	661
Genotype frequency (%):	CC	33.9	23.6	8.5	12	10.2	13.8
	CG	46.4	53.9	40.8	46.3	57.1	48.3
	GG	19.7	22.5	50.7	41.7	32.7	38
p*			**0.104**	0.001	0.001	0.002	0.001
Allelic frequency: (%)	C	57	50.6	28.9	35.2	38.8	37.9
	G	43	49.4	71.1	64.8	61.2	62.1
p**			**0.18**	0.001	0.001	0.002	0.001
p***		0.588	0.529	1	1	0.231	0.563

Chi-square test integrated in HWE software was used for comparative analysis of genotypic and allelic frequencies distribution; p* and p**, *p-values* for genotypic and allelic frequencies distribution (respectively) comparison between respective population and Tanzanian (TNZ); ***p, Fisher’s test *p-value* for deviation of genotypic distribution from HWE. Association was significant if *p<0.05.*

## Discussion

*HLA-G* molecule is mostly reported to be expressed more by tumor cells and exerts immunosuppressive effect to immune cells in variety of ways such as inducing apoptosis, inhibiting cytolytic function of NK cells, inducing the transformation of immune cells to regulatory cells and affecting the immunological behavior of immune cells following trogocytosis (Curigliano et al., 2013). This increased expression of *HLA-G* by tumor cells has thus been explored in context of cancer diagnosis, prognosis and immunotherapy (González et al., 2012; Chen et al., 2010; Kirana et al., 2017; König et al., 2016; Wu et al., 2015).

Our results showed that *sHLA-G* levels were significantly lower in breast cancer patients than that in normal controls. This is so contrary to the most studies’ findings which report the significantly higher amount of *sHLA-G* in breast cancer and other cancer patients than in normal controls (Ugurel et al., 2001; Jeong et al., 2014; Pan et al., 2016; He et al., 2010). It is plausible that our results have been influenced by the medical intervention exerted to breast cancer patients recruited in our study. Most patients were under treatment regimens such as chemotherapy, hormone therapy, radiotherapy, and 81.3% had undergone mastectomy. 

These interventions plausibly affect the bioavailability of *sHLA-G* in circulation. Since tumor cells express more *HLA-G*, it is expected that surgical removal of such cells will subsequently lower the expression levels of *HLA-G* in cancer patients. In this study, we found the significant lower levels of *sHLA-G* in mastectomized patients than in patients who had not yet undergone mastectomy. Our ROC analysis revealed a good discriminatory performance of *sHLA-G* to discern between mastectomized patients and non-mastectomized patients. Also, chemotherapy has been reported to significantly reduce the *sHLA-G* levels among high grade ovarian cancer patients (Rutten et al., 2014).

The corroborative findings on how such medical intervention affect* sHLA-G* bioavailability among breast cancer patients have been reported in the prospective study by Sayed et al., (2010). In this study, the pre-operative *sHLA-G* levels were compared with *sHLA-G* levels 6 months and 12 months later after breast cancer surgery while patients were under adjuvant therapy. There was a significant decrease in *sHLA-G* levels to the extent that, after 6 and 12 months, 66.7% and 97.8% of patients respectively had *sHLA-G* levels below the physiological mean levels of *sHLA-G* observed among the normal controls. Our findings that *sHLA-G* levels are higher in non-mastectomized breast cancer patients than in mastectomized patients, together with the time-dependent decrease in *sHLA-G* levels observed in Sayed et al., (2010) study may be translated into possible reliance on *sHLA-G *as a biomarker for prognosis.

The genetic variations in *3’ UTR* of *HLA-G* gene affect its post-transcriptional regulation. The *+3142G/C *present in this region is determined to affect the stability of *HLA-G mRNA*, with C allele associated with more stable mRNA than G allele. Because *HLA-G* is considered to be pro-tumorigenic immunosuppressive molecule, the C allele which is potent to produce more *HLA-G* protein plausibly should be associated with cancer susceptibility. In this study, however, we could not find significance in association between the *+3142G/C* genotypes and breast cancer. Further, we could not find an influence of the *+3142G/C SNP* on either s*HLA-G* levels or prediction of the metastatic-free survival of breast cancer patients. 

Of course there have been conflicting findings on how +3142G/C polymorphism and some other *3’UTR* genetic variations are associated with various cancers. The variation in genetic structure of *HLA-G 3’UTR *region across different populations may undergird this inconsistency in associations of these genetic variations with a given trait or pathological condition. The different patterns of linkage disequilibrium (LD) between the allelic variants in *HLA-G 3’UTR* across different populations worldwide has been reported by Sabbagh et al., (2014). This difference could be the basis for the existing controversy over the association studies looking for *HLA-G 3’UTR* allelic variants and specific physiological or pathological conditions (Sabbagh et al., 2014). Squaring with this, the significant differences in allelic and genotypic frequencies distributions were observed when our study population was compared with the most African related populations’ *+3142G/C* data from HapMap and 1,000 Genomes databases.

One key limitation of this study is the lack of sufficient number of breast cancer patients who were neither under medication nor had undergone mastectomy. The inclusion of significant size of this group could be crucial in determining unequivocally whether the unexpected low *sHLA-G* levels observed in breast cancer patients are attributable to the medical interventions or otherwise. Before we can fully attribute the unexpected low levels of *sHLA-G* in breast cancer patients to medical intervention, more studies consisting sufficient number of patients who have not yet started medication are emphasized.

To conclude, we have found in this study the significantly lower levels of *sHLA-G* in Tanzanian breast cancer patients compared to normal controls. The results are more likely to have been influenced by the medical interventions exerted to the patients, as mastectomized patients had significantly lower *sHLA-G* levels than non-mastectomized patients. The changes in *sHLA-G *levels in response to medical intervention suggest its potential utility for breast cancer prognosis. More researches comprising sufficient number of naïve breast cancer patients and those already subjected to specific medical interventions are highly recommended to provide unequivocal evidence of *sHLA-G* as a diagnostic and prognostic marker of cancer.

This study also does not provide evidence on association between *+3142G/C* polymorphism and breast cancer, as there was a relatively similar distribution of frequencies of genotypes and alleles between breast cancer patients and normal controls. Similarly, the effect of this *SNP* on *sHLA-G* levels was not found. Since post-translational regulation of *HLA-G* can be affected by multiple genetic variations existing in* 3’UTR*, it is important to study the haplotype blocks associated with *HLA-G* expression and susceptibility to different pathological conditions. 
